# Characterization of Post-Hypoglycemic Hyperglycemia in Children and Adolescents With Type 1 Diabetes: The EPHICA Study

**DOI:** 10.3389/fendo.2022.887976

**Published:** 2022-06-27

**Authors:** Victoria Colinet, Philippe A. Lysy

**Affiliations:** ^1^ Pôle PEDI, Institut de Recherche Expérimentale et Clinique, UCLouvain, Brussels, Belgium; ^2^ Specialized Pediatrics Service, Cliniques universitaires Saint-Luc, Brussels, Belgium

**Keywords:** type 1 diabetes, children, hypoglycemia, hyperglycemia, glycemic variability

## Abstract

**Background:**

In patients with diabetes, the dynamics in which hypoglycemia recovers impacts cardiovascular disease risk. Our study investigated the extents of “post-hypoglycemic hyperglycemia (PHH)” (i.e. hypoglycemia that recover to hyperglycemia in any circumstance) and factors likely to influence PHH characteristics in a pediatric cohort of patients with type 1 diabetes (T1D).

**Methods:**

We collected retrospective continuous glucose monitoring (CGM) data from 142 pediatric patients with T1D to characterize episodes of PHH during a two-month follow-up period. Factors influencing PHH were determined using univariate and multivariate analyses.

**Results:**

In our EPHICA cohort, PHH rate was 0.6 ± 0.3 episode/day and correlated (r=0.33; p<0.0001) with hyperglycemia rate (2.6 ± 0.5 episodes/day). The global proportion of hyperglycemia corresponding to PHH was 0.22 ± 0.1, yet 14.8% of patients had more than 1/3 of hyperglycemia related to PHH. Episodes of PHH lasted 239.6 ± 124.8 minutes with a hyperglycemic peak of 258.8 ± 47.1 mg/dL. Only 12.2% of PHH occurred at night. While a younger age (<12 years) and lower body mass index (BMI) (SDS: -2 to 1.6) were associated with higher daily PHH rates, teenagers (≥12 years) and obese patients experienced longer PHH and higher hyperglycemic peaks. Parameters of glycemic variability (i.e. HbA_1C_, IDAA_1C_ and GTAA_1C_) moderately correlated with PHH duration and related hyperglycemic peak. Multivariate analysis confirmed these results, as factors likely to influence PHH rate were phenotype (age and BMI) and glycemic variability parameters (time in range, mean glycemia, HbA_1C_ and GTAA_1C_).

**Conclusion:**

Our EPHICA study highlights the importance of PHH as a prominent component of hyperglycemia in some children and adolescents with T1D. Factors associated with PHH features are age, BMI and parameters of glycemic control. Young and lean children are more prone to experience hypoglycemia that recover with hyperglycemia, but adolescents and obese children tend to experience hyperglycemia of longer duration.

## Introduction

In patients with type 1 diabetes (T1D), the development of micro- and macrovascular complications depends, at least partly, on glycemic variability (GV) that defines individual degrees of blood glucose variation according to specific indexes (1). While these indexes - e.g. standard deviation (SD), coefficient of variation (CV), or mean amplitude glycemic excursion (MAGE) - cover many aspects of blood glucose fluctuation, they fail to integrate a dynamic estimation of the longitudinal evolution of a particular parameter, as for example the immediate recovery of hyper- or hypoglycemia.

Yet this dynamic estimation is key, especially regarding hypoglycemia, since it is known that the dynamics in which hypoglycemia recovers impacts cardiovascular risk, additionally to pejorative effects of recurrent hypoglycemia *per se* – i.e. endothelial dysfunction, oxidative stress and inflammation (2, 3). Indeed, the study by Ceriello et al. (4), conducted in a small cohort of patients with T1D demonstrated that insulin-induced hypoglycemia that recovered by reaching hyperglycemia were associated with expression of oxidative stress (i.e. nitrotyrosine and 8-iso prostaglandin F2α) and inflammation (i.e. intracellular adhesion molecule-1a and interleukin-6) biomarkers (4). In 2014, the same team demonstrated for the first time the impact of recovery from hypoglycemia with hyperglycemia on the activation of thrombosis (and oxidative stress), both in patients with diabetes and in healthy subjects. Contrarily, the harmful effects of hypoglycemia on thrombosis activation were annihilated when hypoglycemia recovered to normoglycemia. The authors therefore suggested that recovery from hypoglycemia may play an important role on the cardiovascular risk associated with T1D (5).

In Belgium since June 2016, children and adolescents with T1D can benefit from continuous glucose self-monitoring (6), offering a unique possibility for detailed analysis of daily fluctuation of blood glucose levels. Our EPHICA (Evaluation of Posthypoglycemic Hyperglycemia in Children and Adolescents with diabetes) study introduced the concept of “post-hypoglycemic hyperglycemia” (PHH). The concept of PHH corresponds to hypoglycemia that recover to hyperglycemia in any circumstance (e.g. excess of carbohydrate intake during hypoglycemia, lack of insulin coverage, modification of physical activity level) and as such is not related to the controversial Somogyi effect (7). In the EPHICA study, we hypothesized that PHH are a prominent component of hyperglycemia in children and adolescents with T1D. Our study aimed to determine the factors likely to influence the PHH rate, duration and hyperglycemic peak in a pediatric cohort of patients with T1D.

## Material and Methods

### Study Design and Participants

The EPHICA study was designed as a monocentric and retrospective trial to characterize episodes of PHH in children and adolescents with T1D. The study was conducted in the pediatric diabetes center of Cliniques universitaires Saint-Luc (CUSL, Brussels, Belgium). All procedures were conducted in accordance to the Declaration of Helsinki. Study protocol was approved by the Comité d’Ethique Hospitalo-Facultaire of CUSL, 2019/22AOU/359. Patient enrollment and the analysis phase extended from October 17^th^, 2019 to September 21^th^, 2020.

Patients eligible to the study were aged 7 to 18 years, were diagnosed with T1D according to the criteria of the American Diabetes Association (ADA) (8) and were under Freestyle Libre^®^ (Abbott) continuous glucose monitoring (CGM).

The exclusion criteria were an age of less than 7 years; the absence of anti-pancreatic islet autoantibodies; use of medication that may affect insulin secretion and insulin sensitivity; the presence, at inclusion, of celiac disease diagnosed by a pathological duodenal biopsy within one month; the presence, at inclusion, of an autoimmune or autoinflammatory disease other than T1D, or of an active malignant disease; morbid obesity defined by a body mass index (BMI) with a SDS greater than three standard deviations (9); hepatic, renal or adrenal insufficiency; dysmorphia with suspected underlying genetic syndrome; a history of bone marrow allograft or post-hemolytic-uremic syndrome diabetes; participation in another study within the previous three months, with administration of blood derivatives or potentially immunomodulatory treatments. Patients not under CGM, in partial remission phase (*cfr infra*), or with a proportion of CGM data recovered lower than 60%, were also excluded from the study. Also, the study did not include patients under sensor-augmented insulin pump therapy or hybrid closed-loop systems.

### Study Procedures

The retrospective EPHICA study consisted of the analysis of historical data, namely already recorded in the medical file, of an active cohort of 242 patients registered in the pediatric diabetes convention center of the CUSL and under CGM. The retrospective analysis of the available data from these active patients corresponded to a period of minimum one month to maximum six months preceding the analysis phase.

Data collected in the Medical Explorer software using an Excel file version 15.39 were age, sex, height, BMI, date of diagnosis of T1D, presence of diabetic ketoacidosis at diagnosis, insulin treatment regimen. SDS for height and BMI were calculated for age and gender according to reference standards of Roelants et al. (10). The total daily dose of insulin, HbA_1C_ levels and glycemic monitoring readings (by CGM) were defined for three different outpatient visits (each visit spaced approximately by three months) to calculate glycemic target-adjusted HbA_1C_ (GTAA_1C_) (11) and insulin dose-adjusted Hemoglobin A_1C_ (IDAA_1C_) scores (12). Mean HbA_1C_, GTAA_1C_ and IDAA_1C_ were calculated over three outpatient visits. Percentage of normoglycemia or time in range (TIR) and glycemic means were also measured over a time interval of 1 to 3 months. All CGM data were analyzed using R [R Core Team (2021)]. R: A language and environment for statistical computing. R Foundation for Statistical Computing, Vienna, Austria. URL https://www.R-project.org/).

**Figure 1:**
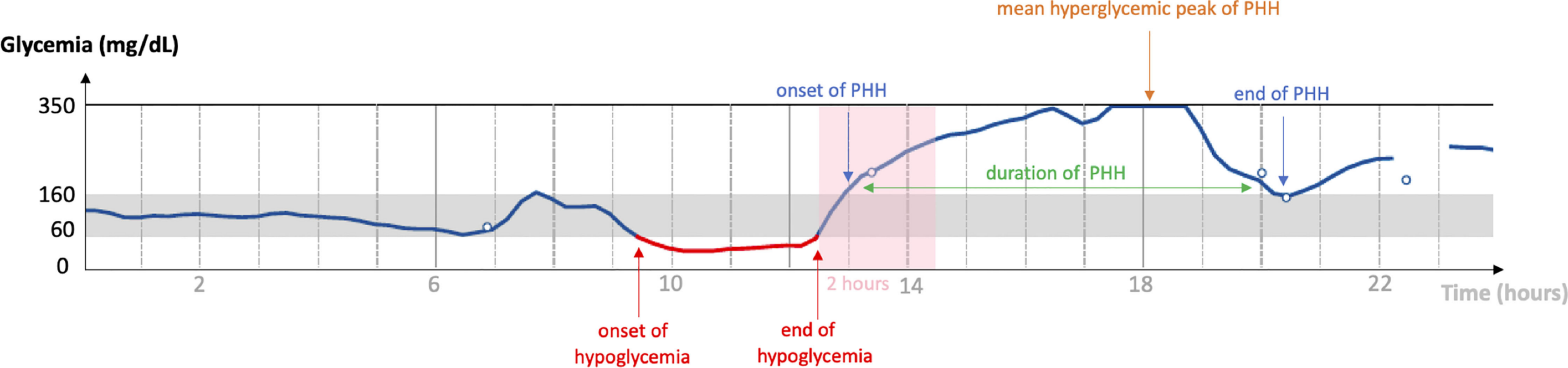
Diagram of a typical PHH. Graphical representation of a continuous analysis of subcutaneous glucose (vertical axis) over a period of 24 hours (horizontal axis). The normoglycemia range is 60-160 mg/dL. In this example: end of hypoglycemia at 12:30 p.m., onset of PHH at 1:00 p.m., PHH hyperglycemic peak at 350 mg/dL between 5:30 and 6:45 p.m., end of PHH at 8:30 p.m. Hyperglycemia occurred less than 2 hours after the end of hypoglycemia; PHH lasted for 7 hours and 30 minutes. PHH: post-hypoglycemic hyperglycemia.

The proportion of PHH was calculated by defining hyperglycemia as a subcutaneous glucose value >160 mg/dL (i.e. above the range of 60-160 mg/dL currently used in Belgian clinical diabetes centers (13, 14) and corresponding to any difference in levels of the CGM curve exceeding the value of 160 mg/dL. Hypoglycemia was defined as a subcutaneous glucose value <60 mg/dL and corresponding to any change in levels of the CGM curve below the value of 60 mg/dL. PHH was defined such as hyperglycemia following a hypoglycemic episode within a period of maximum two hours. The duration of a PHH episode was the time spent between the onset and the end of hyperglycemia. The mean hyperglycemic peak was the highest subcutaneous glucose value during hyperglycemia **(** **Figure 1** **)**.

Factors to be analyzed for their correlation to the mean rate, duration, hyperglycemic peak of PHH and hyperglycemia rate were the patient phenotype (i.e. age, sex, duration of diabetes, BMI, height), characteristics of diabetes (i.e. ketoacidosis at diagnosis, insulin treatment regimen) and parameters of GV (i.e. average of HbA_1C_, GTAA_1C_ and IDAA_1C_ over 3 outpatient visits, TIR and mean glycemia). Discrete variables were subdivided into categories, as shown in **Table S1**. Age, duration of diabetes and BMI were also divided into categories to obtain discrete variables.

### Remission Status

Remission status was determined using the IDAA_1C_ score (15), whose formula is: HbA_1c_ (%) + (4 × insulin dose (U/kg body weight per 24 h). The remission status is defined by a IDAA_1C <_9. We also used the GTAA_1C_ score as follows: HbA_1C_ (%) - (3 × % normoglycemia (70-180 mg/dL)) (11). The remission status is defined by a GTAA_1C_<4.5.

### Statistical Analysis

The statistical significance level used for all analyses was 0.05. Demographic and clinical data are reported as mean ± SD for continuous variables and as numbers and proportions for categorical variables. The level of statistical significance used for all analyzes was 0.05. Demographic and clinical data are reported as mean ± standard deviation for continuous variables and as numbers and proportions for categorical variables. Linear regression was used for continuous variables. The comparisons between groups were made using Student t-test (if two groups) or the analysis of variance “ANOVA” (if more than two groups) or their non-parametric equivalent (respectively Mann-Whitney U test and Kruskal-Wallis test), as appropriate. The correlations were estimated Pearson’s correlation coefficient. Multivariate analysis was performed using multiple linear regression. The statistical methodology was evaluated with Mrs. Céline Bugli (LIDAM, SMCS, UCLouvain) and carried out with the JMP Pro 14.3.0 software. The figures were made using GraphPad Prism software (version 9.1.2).

## Results

### Patient Characteristics

**Figure 2:**
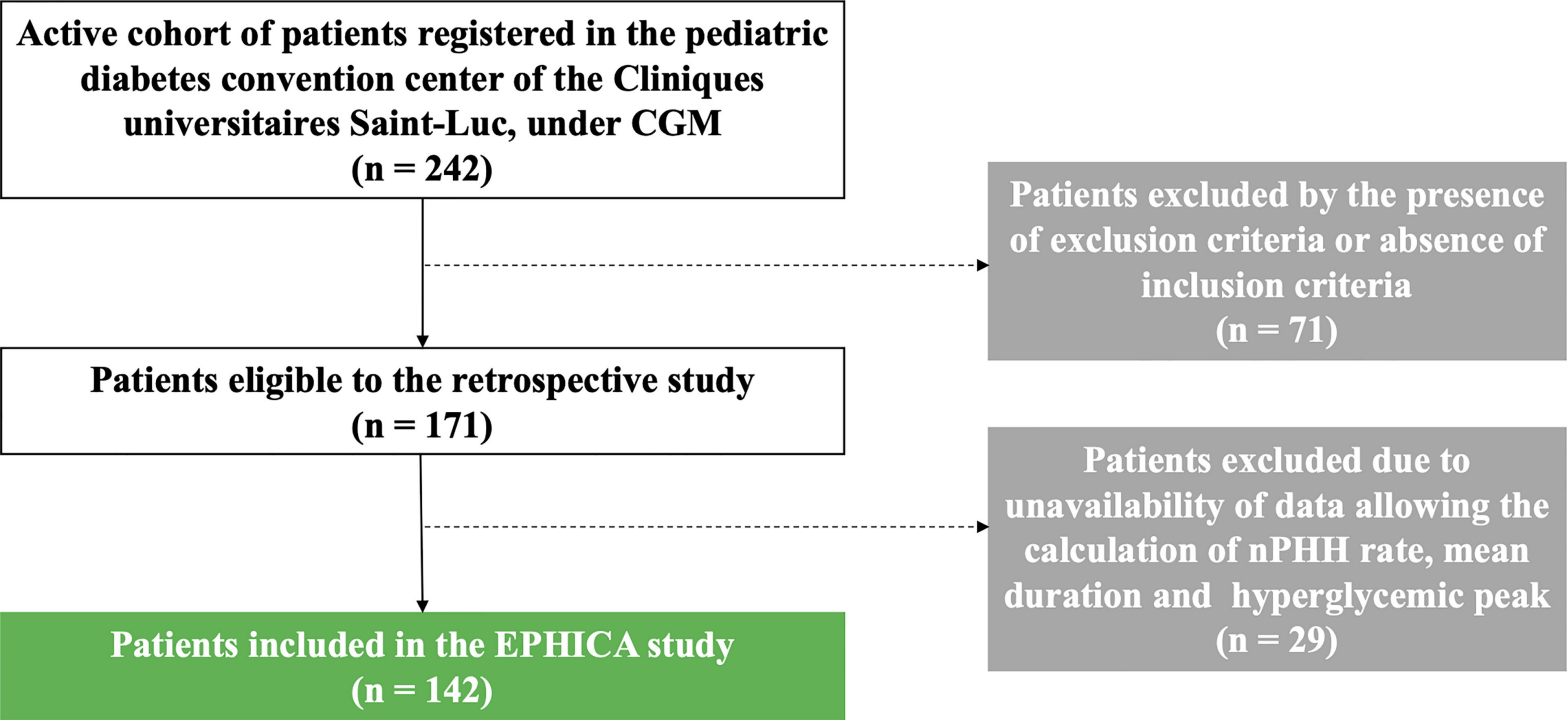
Flow chart of the EPHICA study. Among de 242 patients registered in the pediatric diabetes convention center of the Cliniques universitaires Saint-Luc, 71 patients were excluded by the presence of exclusion criteria or absence of inclusion criteria. Among the 171 patients eligible to the retrospective study, 29 patients were excluded due to unavailability of data allowing the calculation of the PHH rate, the mean duration and the hyperglycemic peak of PHH. Finally, 142 patients were included in the EPHICA study.

In the EPHICA study, we included 142 children and adolescents out of 242 patients (**Figure 2**) registered in the pediatric diabetes convention center of the Cliniques universitaires Saint-Luc. Clinical characteristics of the cohort are presented in **Table 1**. Mean age was 13.0 ± 2.9 years. Proportion of male subjects was 49.3% (70/142) and mean diabetes duration was 5.6 ± 3.6 years. Mean height SDS was -0.1 ± 1.0 and mean BMI SDS was 0.5 ± 1.1. Proportions of ketoacidosis and ketosis at diagnosis were 37.5% (45/142) and 72.4% (84/142), respectively. The majority of patients were under five daily insulin injection regimen (67.6%) while a minority was on morning-evening insulin injection regimen (14.1%) or were under pump therapy (18.3%). In the study cohort, mean HbA_1C_ level was 7.4 ± 0.8%, mean IDAA_1C_ score was 11.1 ± 1.6, mean GTAA_1C_ score was 6.0 ± 1.1, mean percentage of normoglycemia was 43.6 ± 12.1%, and mean glycemia was 160.3 ± 28.3 mg/dL.

**Table 1:** Characteristics of the EPHICA cohort.

Phenotype	
Age – years	13.01 ± 2.86
Gender	
Boys	70 (49.3)
Girls Duration of diabetes – years	72 (50.7) 5.63 ± 3.61
Height – SDS	-0.1 ± 1.02
BMI – SDS	0.49 ± 1.11
**Characteristics of diabetes**	
Acidosis at diagnosis	
Yes	45 (37.5)
No	75 (62.5)
Ketosis at diagnosis	
Yes	84 (72.41)
No	32 (27.59)
Insulin treatment regimen	
Five injections	96 (67.61)
Morning-Evening	20 (14.08)
Pump	26 (18.31)
**Glycemic variability parameters**	
Mean HbA_1C_	7.38 ± 0.78
Mean IDAA_1C_	11.11 ± 1.58
Mean GTAA_1C_	6.03 ± 1.06
Percentage of normoglycemia (TIR)	43.56 ± 12.13
Mean glycemia	160.28 ± 28.3

SDS, standard deviation score; TIR, time in range.

Data expressed as mean ± SDS or n (%).

### Description of Hyperglycemia and PHH

**Figure 3:**
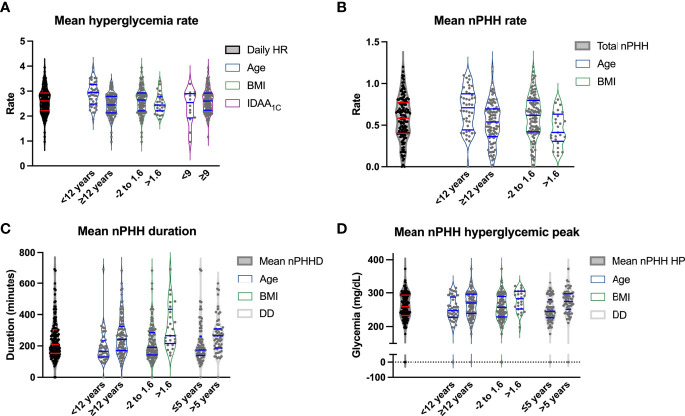
Relations between cohort characteristics, diabetes parameters, and features of PHH. Violin plots representing in the vertical axis mean hyperglycemic rate **(A)**, PHH rate **(B)**, PHH duration **(C)** and PHH hyperglycemic peak **(D)** according to age <12 versus ≥12 years, BMI between -2 and 1.6 versus >1.6, mean IDAA_1C_<9 versus ≥9 **(A)**, and diabetes duration ≤5 versus >5 years **(C**, **D)**. The first plot corresponds to the data range of the vertical variable. The horizontal blue lines correspond to the median and 95% confidence intervals. HR, hyperglycemic rate; BMI, body mass index; PHH, post-hypoglycemic hyperglycemia; PHHD, PHH duration; DD, diabetes duration; PHH HP, PHH hyperglycemic peak.

In the whole cohort, the mean daily rate of hyperglycemia (>160 mg/dL) was 2.6 ± 0.5 episodes/day (0.9; 3.9) (**Figure 3A**). Patients experienced a mean daily rate of PHH of 0.6 ± 0.3 episode/day (0; 1.2) (**Figure 3B**). The correlation between PHH rate and hyperglycemic rate was weak but significant (r= 0.33; p<0.0001). Episodes of PHH lasted 239.6 ± 124.8 minutes in average (0; 692.9) (**Figure 3C**), with a mean hyperglycemic peak of PHH being measured at 258.8 ± 47.1 mg/dL (0; 373) (**Figure 3D**). We researched the time of onset of PHH among randomly selected 40 patients divided into 4 groups according to their PHH levels. **Supplemental Figure 3B** show that few hypoglycemia occurred at night (total of 12.2% during nighttime, i.e. 10:00 p.m. – 07:00 a.m.) and that PHH were spread across daytime. Among the 142 patients, 7.7% presented a negligible level of PHH, i.e. ≤0.14 PHH/day, while 21.8% presented a significant level of PHH, i.e. ≥0.8 PHH/day. The mean proportion of hyperglycemia corresponding to PHH (ratio PHH rate/hyperglycemia rate) was 0.22 ± 0.1. In addition, 14.8% of patients had more than 1/3 of their hyperglycemia corresponding to PHH. The correlation between PHH rate and hyperglycemic rate was weak but significant (r= 0.33; p<0.0001). When analyzed using a target glycemic range of 70-180 mg/dL, results were comparable to the ones obtained using our 60-160 mg/dL range both in terms of frequency (0.52 ± 0.31, p=0.45) and duration (239 ± 277 minutes, p=0.84) of PHH.

### Influence of Patient Phenotype, Diabetes Characteristics and Glycemic Regulation on PHH and Hyperglycemia

To unravel the characteristics of our patient cohort that correlated with the appearance of PHH, we performed univariate and multivariate analyses using defined parameters (**Table S1**).

In univariate analysis (**Table 2**), an age of <12 years was significantly associated with higher hyperglycemia and PHH rates (p<0.0001 and p=0.0011, respectively; **Figures 3A, B**), while an age ≥12 years was associated with higher PHH duration and hyperglycemic peak (p=0.0004 and p=0.04, respectively; **Figures 3C, D**). Patients with a diabetes duration >5 years had higher levels of PHH duration compared to patients with a diabetes duration of diabetes <2 years or between 2 and 5 years (p=0.0005). In addition, patients with a diabetes duration of <2 years had significantly lower PHH hyperglycemic peak compared to patients with diabetes duration between 2 and 5 years or >5 years (p=0.005). A BMI SDS between -2 and 1.6 was associated with a higher PHH rate (p=0.015; **Figure 3B**), while a BMI SDS >1.6 was significantly associated with higher duration and hyperglycemic peak of PHH (p=0.0003 and p=0.01, respectively; **Figures 3C, D**). An insulin pump therapy was significantly associated with a higher hyperglycemia rate compared to treatment by five injections (p=0.0001), while a regimen “morning-evening” was significantly associated with a higher PHH rate compared to treatment by five injections (p=0.03). Among parameters of diabetes control, we observed that TIR moderately correlated with PHH duration (r=-0.65; p<0,0001) and hyperglycemic peak (r=-0.7; p<0,0001). Mean glycemia correlated weakly with PHH rate (r=-0.35; p<0,0001) but moderately with PHH duration (r=0.75; p<0,0001) and hyperglycemic peak (r=0.65; p<0,0001). Also, HbA_1C_, IDAA_1C_ and GTAA_1C_ moderately correlated with PHH duration (r=0.55, 0.45 and 0.6, respectively; p<0,0001) and hyperglycemic peak (r=0.6, 0.55 and 0.65, respectively; p<0,0001). Regarding these parameters, only IDAA_1C_ weakly correlated with hyperglycemia rate (r=0.15, p<0,0001; **Figure 3A**).

**Table 2:** Univariate analysis of parameters influencing mean PHH rate, duration and hyperglycemic peak, and mean hyperglycemia rate.

Mean hyperglycemia rate	Versus	r	p value	β
*Discrete variables: factors associated with a higher mean hyperglycemic rate*				
Age [7;12]	[12;18]		< 0.0001	-1.889
ITR Pump	5 Injections		0.0001	
*Continuous variables*				
Mean IDAA_1C_		0.15	0.0465	-0.5214
**Mean PHH rate**				
*Discrete variables: factors associated with a higher mean PHH rate*				
Age [7;12]	[12;18]		0.0011	-2.511
BMI SDS [-2;1.6]	> 1.6		0.0152	-0.9386
ITR Morning-Evening	5 Injections		0.0287	
*Continuous variables*				
Mean glycemia		-0.35	< 0.0001	-38.59
**Mean PHH duration**				
*Discrete variables: factors associated with a higher mean PHH duration*				
Age [12;18]	[7;12]		0.0004	0.0069
Duration of diabetes > 5	< 2		0.0005*	0.0085^#^
> 5	[2;5]			
BMI SDS > 1.6	[-2;1.6]		0.0003	0.0026
*Continuous variables*				
Percentage of normoglycemia (TIR)		-0.65	< 0.0001	-0.0621
Mean glycemia		0.75	< 0.0001	0.1723
Mean HbA_1C_		0.55	< 0.0001	0.0036
Mean IDAA_1C_		0.45	< 0.0001	0.0059
Mean GTAA_1C_		0.6	< 0.0001	0.0052
**Mean PHH hyperglycemic peak**				
*Discrete variables: factors associated with a higher mean PHH hyperglycemic peak*				
Age [12;18]	[7;12]		0.04	0.0106
Duration of diabetes [2;5]	< 2		0.005*	0.02616^#^
> 5	< 2			
BMI SDS > 1.6	[-2;1.6]		0.01	0.0049
*Continuous variables*				
Percentage of normoglycemia (TIR)		-0.7	< 0.0001	-0.1856
Mean glycemia		0.65	< 0.0001	0.4038
Mean HbA_1C_		0.6	< 0.0001	0.0099
Mean IDAA_1C_		0.55	< 0.0001	0.0186
Mean GTAA_1C_		0.65	< 0.0001	0.0152

ITR, Insulin treatment regimen; SDS, standard deviation score; TIR, time in range. *Post-hoc ANOVA analysis. ^#^β coefficient of “duration of diabetes” during univariate analysis.

**Figure 4:**
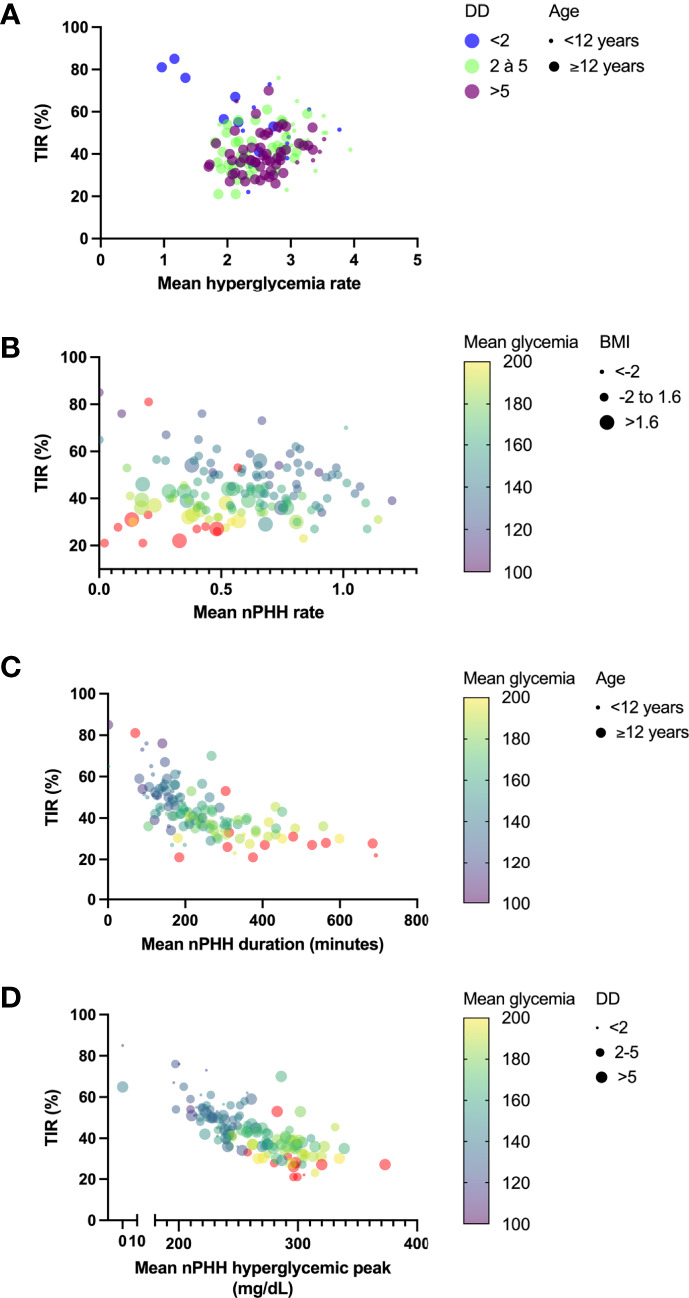
Principal determinants of PHH main characteristics. Graphs represent principal determinants of hyperglycemia rate **(A)**, PHH rate **(B)**, PHH duration **(C)** and PHH hyperglycemic peak **(D)**. **(A)** Relations between TIR and hyperglycemia rate were decomposed into diabetes durations (dots with color code) and age (dot size). Although TIR did not correlate with hyperglycemia rate in multiple linear regression analysis (p=0.24), it was represented in y axis for homogenization across the graphs. **(B)** Relations between TIR and PHH rate were decomposed into mean glycemia (dots with continuous colored purple-yellow gradient) and BMI (dot size). **(C)** Relations between TIR and PHH duration were decomposed into mean glycemia (dots with continuous colored purple-yellow gradient) and age (dot size). **(D)** Relations between TIR and hyperglycemic peak were decomposed into mean glycemia (dots with continuous colored purple-yellow gradient) and diabetes duration (dot size), although this later parameter did not significantly correlate with mean hyperglycemic peak (p=0.09). TIR, time in range; DD, diabetes duration; PHH, post-hypoglycemic hyperglycemia; BMI, body mass index.

In multivariate analysis (**Table 3**; **Figure 4**), parameters which taken together were correlated with hyperglycemia rate were age (p<0.0001) and diabetes duration (p=0.007). Parameters which correlated with PHH rate were age (p=0.01), BMI (p<0.0001), TIR (p=0.007), mean glycemia (p<0.0001), HbA_1C_ (p=0.025) and GTAA_1C_ (p=0.02). Also, parameters which correlated with PHH duration were age (p=0.04), TIR (p=0.035) and mean glycemia (p<0.0001), while only TIR (p=0.0003) and mean glycemia (p=0.015) correlated with PHH hyperglycemic peak. Furthermore, the correlation between age and BMI was weak but significant (r= 0.23; p=0.007).

**Table 3:** Multivariate analysis of parameters influencing mean PHH rate, duration and hyperglycemic peak, and mean hyperglycemia rate.

Mean hyperglycemia rate	p value
*Discrete variables*	
Age	<0.0001
Duration of diabetes	0.007
**Mean PHH rate**	
*Discrete variables*	
Age	0.01
BMI SDS	<0.0001
*Continuous variables*	
Percentage of normoglycemia (TIR)	0.007
Mean glycemia	<0.0001
Mean HbA_1C_	0.025
Mean GTAA_1C_	0.02
**Mean PHH duration**	
*Discrete variables*	
Age	0.04
*Continuous variables*	
Percentage of normoglycemia (TIR)	0.035
Mean glycemia	<0.0001
**Mean PHH hyperglycemic peak**	
*Continuous variables*	
Percentage of normoglycemia (TIR)	0.0003
Mean glycemia	0.015

TIR, time in range.

## Discussion

Our EPHICA study highlights the importance of PHH as a major component of hyperglycemia in some children and adolescents with T1D. Indeed, the correlation between the level of PHH and hyperglycemia is present, the proportion of hyperglycemia corresponding to PHH is 22% on average in the cohort, and 14.8% of patients had more than 1/3 of their hyperglycemia which corresponded to PHH.

Our study reveals that factors influencing PHH features (rate, duration and hyperglycemic peak) in a pediatric cohort of patients with T1D are age, BMI and parameters of glycemic control. A younger age (<12 years) was associated with higher mean hyperglycemia and PHH rates while adolescents (≥12 years) experienced higher mean PHH duration and hyperglycemic peak during PHH. A lean mass (BMI SDS between -2 and 1.6) was correlated to higher mean PHH rate, while obese subjects (BMI >1.6) had higher PHH duration and hyperglycemic peaks. In multivariate analysis, factors likely to influence mean hyperglycemia and PHH rates were phenotype (age and diabetes duration or BMI) for both and glycemic variability parameters (TIR, mean glycemia, mean HbA_1C_ and mean GTAA_1C_) for PHH rate. Mean PHH duration and hyperglycemic peak were influenced by glycemic variability parameters (TIR and mean glycemia) for both and by phenotype (age) for duration.

Results from our cohort suggest that a younger age (<12 years) and lower BMI (SDS: -2 to 1.6) are significantly associated with higher mean daily hyperglycemia and PHH rates. These young and lean patients have better diabetes control, according to GV parameters (i.e. TIR, mean glycemia, mean HbA_1C_ and GTAA_1C_). Conversely, teenagers (≥12 years) and obese patients experience less PHH but of longer duration and higher amplitude. Our results thus suggest that young and lean children are more prone to experience hypoglycemia that recover with hyperglycemia, while adolescents and obese children tend to experience hyperglycemia of longer duration.

Whereas our study highlights the importance of PHH and the influence of age and BMI in its occurrence, our results show that the PHH rate taken alone does not account *per se* for the balance of diabetes, unlike its duration and amplitude. An action aimed at limiting the duration and the hyperglycemic peak of PHH will have a considerable impact on the balance of diabetes. We defend the hypothesis that these PHH are due to inadequate re-sugaring. During hypoglycemia, lean children (<12 years) might receive lower amounts of carbohydrates than adolescents and/or obese children because, on the one hand, they depend more on third parties (ex: parents) for their care (16), and on the other hand, being lean, they likely consume fewer amounts of carbohydrates compared to older and/or obese patients (17, 18).

Several studies revealed the harmful effects of hypoglycemia recovering by hyperglycemia. However, at present, there is no other study that has evaluated the factors influencing the occurrence, duration and amplitude of PHH in children with T1D. On February 5^th^, 2022, the yield of published studies was of 513 in Pubmed and 164 in Cochrane Library by introducing the following parameters: “diabetes mellitus type 1” AND “hyperglycemia” AND “hypoglycemia” AND “child”. By adding the keyword “glycemic variability”, the yield was of 49 in Pubmed and of 25 in Cochrane Library. None of these studies thoroughly investigated the PHH phenomenon in pediatrics. However, there is a whole body of reports concerning the Somogyi effect, which depicts the body’s spontaneous reaction to late evening hypoglycemia by producing counter-regulatory hormones that induce hyperglycemia in the early morning. Whether or not the Somogyi effect is important in pediatrics is still debated (7, 19). Nevertheless, our study shows that PHH were not only observed at night, but throughout the nychthemere (i.e. full day period). Also, several studies evaluated the presence of rebound hyperglycemia (i.e. hyperglycemia following hypoglycemia) in patients with diabetes, under CGM (20, 21). Yet, the objectives of these studies performed in adult cohorts were to evaluate the influence of predictive alarms or insulin suspension in the occurrence of rebound hyperglycemia, in selected patients [e.g. patients with problematic hypoglycemia in the study by Acciaroli et al. (20)].

The strength of our study is the thorough description of PHH in a cohort of patients representing the clinical reality of long-term type 1 diabetes during childhood. One of the greatest limitations is the retrospective setting of our research and the small sample size of our cohort. Our data would need to be confronted to a larger study group.

Given the nychthemeral timing of PHH, we believe that PHH are largely related to food intake. An extension of our EPHICA study would be to assess the impact of a change in re-sugaring protocol on the PHH duration and hyperglycemic peak. A prospective longitudinal study was designed by our group and consists of the following protocol: a) estimation of individual re-sugaring protocols used by patients and notification of the characteristics of diabetes (insulin injections, resugar, physical activity, symptoms of hypoglycemia, ingested carbohydrates) on a monitoring grid, b) analysis of CGM data and monitoring grid during a two-week period, c) establishment of improved re-sugaring protocols for each patient, and d) analysis of a new monitoring grid and the effects of the individualized protocols on glycemic control during another two-week period. Our team is currently working on the building of electronic tools to overcome the difficulties in retrieving precise data from our pediatric patients in this prospective part of our study. This prospective study will in addition to assess the impact of insulin coverage and level of physical activity on the occurrence of PHH.

## Data Availability Statement

The raw data supporting the conclusions of this article will be made available by the authors, without undue reservation.

## Ethics Statement

The studies involving human participants were reviewed and approved by Comité d’Ethique Hospitalo-Facultaire of CUSL, 2019/22AOU/359. Written informed consent to participate in this study was provided by the participants’ legal guardian/next of kin.

## Author Contributions

VC and PL designed the research and wrote the manuscript. VC collected the research data. All authors contributed to the article and approved the submitted version.

## Funding

This work was supported by Fonds National de la Recherche Scientifique and Belgian Society for Pediatric Endocrinology and Diabetology.

## Conflict of Interest

The authors declare that the research was conducted in the absence of any commercial or financial relationships that could be construed as a potential conflict of interest.

## Publisher’s Note

All claims expressed in this article are solely those of the authors and do not necessarily represent those of their affiliated organizations, or those of the publisher, the editors and the reviewers. Any product that may be evaluated in this article, or claim that may be made by its manufacturer, is not guaranteed or endorsed by the publisher.
