# Supplementary material for: Characterization of Post-Hypoglycemic Hyperglycemia in Children and Adolescents With Type 1 Diabetes: The EPHICA Study
Source: Front Endocrinol (Lausanne). 2022 Jun 27;13:887976. doi: 10.3389/fendo.2022.887976 (PMC9272988; doi:10.3389/fendo.2022.887976)
Supplement: Supplementary file 1 [file DataSheet_1.docx]

**Supplemental data**

**Characterization of post-hypoglycemic hyperglycemia (PHH) in children and adolescents with type 1 diabetes: the EPHICA study**

Victoria Colinet M.D.^1,2^, Philippe A. Lysy Ph.D.^1,2^.

**Supplemental Table 1. Variables evaluated for uni- and multivariate analysis, with categories for discrete variables and age, duration of diabetes and BMI.**

**Supplemental Figure 3B: distribution of PHH during the nychthemere.**

**Tables**

**Supplemental Table 1. Variables evaluated for uni- and multivariate analysis, with categories for discrete variables and age, duration of diabetes and BMI.**

The left column presents the variables to be correlated to the mean hyperglycemia rate and PHH rate, duration and hyperglycemic peak. The right column describes the subdivision into categories of discrete variables. The other variables are continuous.

| **VARIABLE  Phenotype** | **CATEGORIES** |
| --- | --- |
| Age – years  Gender   Duration of diabetes – years  Height – SDS  BMI – SDS | < 12 ; ≥ 12  Boys (B) ; Girls (G)  < 2 ; 2 to 5 ; > 5   < -2 ; -2 to 1,6 ; > 1,6 |
| **Characteristics of diabetes** |  |
| Acidosis at diagnosis  Ketosis at diagnosis  Insulin treatment regimen | Yes ; No  Yes ; No Five injections ; Morning-Evening ; Pump |
| **Glycemic variability parameters** |  |
| Mean HbA_1C_  Mean IDAA_1C_  Mean GTAA_1C_  Percentage of normoglycemia (TIR)  Mean glycemia |  |

SDS: standard deviation score; TIR = time in range.

**Supplemental Figure 3B: distribution of PHH during the nychthemeron.**


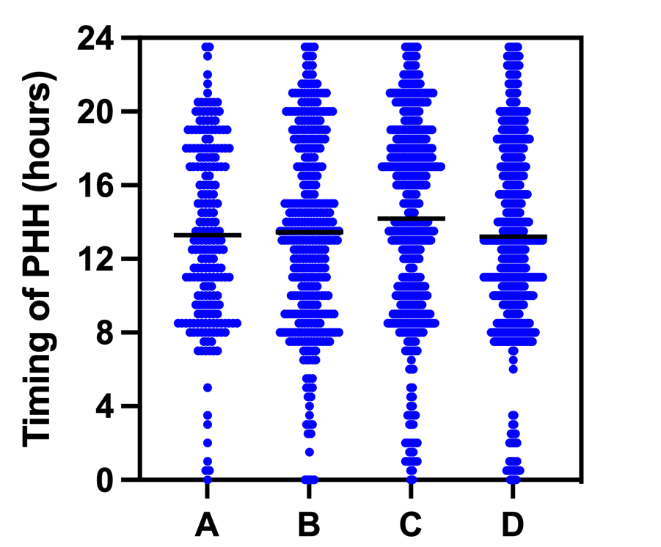
We researched the time of onset of PHH among 40 patients in our cohort, divided into 4 groups according to their PHH rate as follows: group A [0.2- 0.4]; B ]0.4-0.6]; C ]0.6-0.9]; D ]0.9-1.2]. Black bars represent means.
